# Effects of a DNA and multivalent oil-adjuvanted vaccines against pancreas disease in Atlantic salmon (*Salmo salar*) challenged with salmonid alphavirus subtype 3

**DOI:** 10.1016/j.fsirep.2022.100063

**Published:** 2022-08-10

**Authors:** Ragnar Thorarinsson, Jeffrey C. Wolf, Makoto Inami, Hilde Sindre, Eystein Skjerve, Øystein Evensen, Espen Rimstad

**Affiliations:** a Elanco Animal Health, Solheimsgaten 18, Bergen N-5058, Norway; b Experimental Pathology Laboratories Inc., 45600 Terminal Drive, Sterling, VA 20166, United States; c VESO Vikan, Beisvågveien 107, Vikan, Namsos N-7810, Norway; d Norwegian Veterinary Institute, Arboretveien 57, Ås N-1433, Norway; e Norwegian University of Life Sciences, School of Veterinary Medicine, Oluf Thesens vei 22, Ås N-1433, Norway

**Keywords:** Pancreas disease, Salmonid alphavirus, Atlantic salmon, DNA vaccine, Protective immunity

## Abstract

Salmonid alphavirus (SAV) causes pancreas disease (PD) in Atlantic salmon (*Salmo salar*). In seawater-farmed salmonids in the southern part of Norway SAV subtype 3 (SAV3) is dominating. PD continues to cause significant economic and fish health concerns in this region despite years of extensive use of oil-adjuvanted vaccines (OAVs) containing inactivated whole virus (IWV) antigens. In the current study, three commercially available PD vaccines were tested. Group A got a DNA vaccine (DNAV) injected intramuscularly (i.m.) plus an OAV without a PD component injected intraperitoneally (i.p.). Groups B and C got different OAV IWV vaccines injected i.p., respectively. The control group was i.p. injected with saline. Approximately 12 weeks after vaccination, the post smolt groups were challenged in seawater with SAV3 by cohabitation. Samples were collected pre-challenge, and at 19, 54 and 83 days post-challenge (dpc). There were no differences in growth or visible intraperitoneal side effects between the immunized groups prior to challenge. Fish in group A had significantly higher SAV3 neutralizing antibody titers than the other groups pre-challenge and significantly lower SAV3 viremia levels than the control group at 19 dpc. Fish in group A had significantly more weight gain than the other groups measured at 54 and 83 dpc. Prevalence and severity of heart necrosis at 19 dpc and loss of exocrine pancreas tissue at 54 and 83 dpc were significantly lower in groups A and B compared to group C and controls. The cumulative mortality in the control group during the challenge period was 10.5%. Group A experienced the lowest mortality (6.4%) albeit not statistically different from the controls. The results suggest that DNAV may reduce the clinical and economic impact of PD by improved protection against SAV3-induced changes in pancreas tissue and growth impairment.

## Highlights

• Efficacy of a DNA- and conventional vaccines against pancreas disease is compared.
• Higher neutralization antibody levels in the DNA vaccine group compared to controls.
• Significantly lower viremia levels in the DNA vaccine group than the controls.
• Efficacy against disease-induced growth loss and damage in target organs is shown .
• Mortality levels low and not significantly different from the control group.

## 1 Introduction

Pancreas disease (PD) causes serious economic and disease problems in farmed Atlantic salmon (*Salmo salar*) and rainbow trout (*Oncorhynchus mykiss*) in seawater in Norway, Scotland and Ireland [1]. PD is caused by salmonid pancreas disease virus (SPDV), also commonly named salmonid alphavirus (SAV). Six different subtypes of SAV (SAV1-SAV6), have been described based on the nucleic acid sequences encoding the E2 glycoprotein and the nonstructural protein nsP3 [2]. A cross-neutralization study demonstrated close serological relatedness between all the SAV subtypes, with a possible exception for SAV6 [3]. All SAV sub-types except SAV3 have been identified in Scotland and Ireland. Outbreaks of PD caused by SAV3 have to date only been detected in Norway [4] with its distribution limited to the southern coast [1,5]. PD caused by SAV2 is also present in Norway with enzootic distribution largely limited to the mid-region of the coastline [1,6].

Clinical signs of PD include mortality [7,8], reduced growth rates [9,10] and reduced filet quality at slaughter [11]. Histopathological findings include myocarditis and pancreatitis with loss of exocrine pancreatic tissue, and myositis in both red and white skeletal muscle [12,13]. A study of the  use of a commercially available oil-adjuvanted PD vaccine between 2007 and 2009 in the SAV3 enzootic area concluded with the achievement of some reduction in PD prevalence and severity [9]. However, despite extensive use of OAVs with a PD component in the SAV3 area, PD has continued to cause significant animal health problems and economic losses [14]. In 2017, the EU Commission issued a marketing authorization for a DNA vaccine (DNAV) against PD in Atlantic salmon (CLYNAV™, Elanco Animal Health), that was first used commercially for the 2018 Atlantic salmon smolt generation. The primary aim of the present study was to compare the protective effects of the DNAV and multivalent oil-adjuvanted IWV PD vaccines using a SAV3 cohabitation challenge in seawater. The test groups mimicked the different vaccination practices commonly used by the Norwegian salmon farming industry. The design of the efficacy criteria employed in this study mirrors another study published in 2021 that was carried out in parallel where the DNAV and an oil-adjuvanted IWV PD monovalent vaccines were assessed without the concurrent use of a multivalent OAV [15].

## 2 Materials and methods

### 2.1 Fish and vaccination

The study was performed using Atlantic salmon (Stofnfiskur Optimal strain), reared from hatching at the VESO Vikan hatchery (N-7819 Fosslandsosen, Norway). Prior to enrolment, fish were screened immunologically and then transferred to the experimental test facility at VESO Vikan (Namsos, Norway). All tested fish were confirmed negative for antibodies against *Aeromonas salmonicida, Vibrio salmonicida, V. anguillarum* serotype O1 and O2, *V. ordalii, M. viscosa* and infectious pancreatic necrosis virus (IPNV).

Healthy parr were size-graded and anaesthetized with metacain (Finquel vet., ScanVacc) before being intraperitoneally (i.p.) inserted with passive integrated transponder (PIT) tags and registered into VESO Vikan's database. Two weeks later, fish were again anaesthetized, and length and weight were individually recorded. The fish were immunized by injection according to product label specifications using different commercially available vaccine combinations, including PD components used in the Norwegian salmon industry, or injected with sterile saline as negative controls (Saline). At the same time using a separate tank (Tank 2), additional fish were adipose fin-clipped (AFC) to later serve as SAV3 shedders, or as non-vaccinated and non-challenged negative controls (NVNC) for the histopathological analysis. The experimental design and links to the summary of product characteristics (SPC) of the vaccines are outlined in Table 1.

**Table 1:** Treatment groups including group ID's, routes of administration, dose per fish and number of fish per tank.

**Treatment groups (Group ID)**	**Route**	**Dose (ml)**	**No. fish per treatment**	
			**Tank 2**	**Tank 1**
Clynav^a^ + Pentium Forte Plus^a^ (Group A)	i.m. + i*.*p.	0.05 + 0.1	50	111
AJm 1 PD^b^ + AJm 6^b^ (Group B)	i.p.	0.05 + 0.05	50	112
Aquavac PD7^c^ (Group C)	i.p.	0.1	50	112
Physiological saline (Saline)	i.p.	0.05	50	340
No treatment (Naïve)^d^	n.a.	n.a.	200	–

a Produced by Elanco Animal Health. Clynav contains pUK-SPDV-poly2#1 DNA plasmid coding for SPDV proteins. See SPC (https://www.ema.europa.eu/en/documents/product-information/clynav-epar-product-information_en.pdf). Pentium Forte Plus is an OAV containing inactivated *Aeromonas salmonicida* subsp. *salmonicida, Aliivibrio salmonicida, Listonella anguillarum* serotype O1, L. *anguillarum* serotype O2a, *Moritella viscosa* and infectious pancreatic necrosis virus (IPNV). See SPC available in Norwegian only (https://www.legemiddelsok.no/_layouts/15/Preparatomtaler/Spc/07–4936.pdf).

b Produced by Pharmaq. “AJm 1 PD” = ALPHA JECT micro 1-PD is an OAV containing formeldahyde inactivated culture of SPDV. See SPC (https://vmd.defra.gov.uk/productinformationdatabase/files/SPC_Documents/SPC_916517.PDF). “AJm 6″ = ALPHA JECT micro 6, an OAV containing similar antigenic components as Pentium Forte Plus. See SPC (https://vmd.defra.gov.uk/productinformationdatabase/files/SPC_Documents/SPC_1566772.PDF).

c Produced by MSD Animal Health. Aquavac PD7 is an OAV containing inactivated SPDV and similar bacterial components as in AJm6 also inactivated. See SPC available in Norwegian only (https://www.legemiddelsok.no/_layouts/15/Preparatomtaler/Spc/13–9717.pdf).

d Naïve fish where adipose fin clipped for easy identification used as shedders (*n* = 170) and as NVNC controls in the histopathologic evaluation. “i.m.” = intramuscular, “i.p.” = intraperitoneal, “n.a.” = not applicable.

### 2.2 Husbandry, feeding and smoltification

Fish were maintained at 12 ± 1 °C throughout the study in two 1.5 m diameter tube overflow system tanks denoted as Tank 1 and Tank 2 with flow rates adjusted so that oxygen saturation levels near the outlet remained ≥70%. Cleaning of the tanks and removal of dying and dead fish occurred daily. Feeding was stopped at a minimum of 24 h prior to handling or sampling of fish. Fish were kept sedated using AQUI-S VET (isoeugenol, MSD Animal Health) during each sampling according to the product's label specifications to minimize stress. Euthanasia of fish during the sampling process, and when removing moribund and terminally diseased animals, was performed using an overdose of benzocaine chloride. Fish were fed standard commercial extruded pellets (Skretting) throughout the study. Post vaccination, fish were fed *ad libitum* for 36 days, and thereafter at 2% body weight per day until challenge. The feeding rates were restored to *ad libitum* levels throughout the challenge period. Fish were exposed to 12 h light and 12 h darkness (12:12) for 6 weeks followed by continuous 24 h light exposure (24:0) for another 6 weeks prior to being transferred to seawater (salinity maintained at 32 ± 3‰). All handling of fish in the study was carried out in accordance with Norwegian “Regulation on Animal Experimentation” and the study protocol was approved by the Norwegian Animal Research Authority before initiation (FOTS ID14276).

### 2.3 Weight and length gain, side effects, blood sampling and neutralization test

The weights and fork lengths of all the PIT-tagged fish enrolled in this study were recorded and scanned at the time of vaccination into a database as described in Section 2.1. After an immunization period of 1030° days (dd), all fish in Tank 2 except the naïve AFC ones where euthanized and sampled as detailed in Table 2. For each fish, the PIT-tag ID was scanned again into the database so that all parameters measured could thereafter be traced to individual fish. Weight and fork length measurements were again recorded followed by blood collection from the caudal vein using heparin-coated vacutainers that were placed immediately into crushed ice. After centrifugation at 1000 x g for 10 min, plasma samples were retrieved and stored at −80 °C until use. Autopsy examination was conducted in a blinded manner and vaccine-induced abdominal adhesions graded using a progressive scoring system from 0 (no adhesions) to 6 as previously described [16].

**Table 2:** Overview of sampling objectives and time points from Tank 2 (pre-challenge after 1030 dd) and Tank 1 (post challenge) including number of fish for each of the 4 groups (Groups A, B, C and Saline).

**Sampling objective**	**Number of fish per group**			
	**Tank 2**	**Tank 1**		
	**Non-infected**	**19 dpc**	**54 dpc**	**83 dpc**
Length and weight	50^a^	20^a^	20 a	Survivors^b^
Side effects i.p.	30^c^			
Neutralization test	20^d^			
Histopathology	–	20	20	20
Viremia	–	20	–	–

a The same fish used for the different samples taken as shown in the rows below.

b The first 20 fish per group also used for histology. The length and weight of the survivors excluding the shedders.

c Entailed the first 30 fish sampled.

d Entailed the first 20 fish sampled.

The neutralization test was performed as previously described [17] with some modifications. In short, two-fold dilution series of plasma specimens were incubated with SAV3 (Isolate 4 from Taksdal et al. [10], GenBank LT630447) for 2 h and then seeded with CHSE-214 cells in 2 replicate wells (96 well plate). After 3–4 days of incubation at 15 °C, the cell layer was fixed using 80% acetone. SAV-infected cells were visualized using an indirect immunofluorescence test according to the procedure described by Falk et al. [18], but with the use of monoclonal antibody 17H23 directed against the E2 glycoprotein of SAV [19] as the primary antibody and with biotin labelled goat anti-mouse Ig and FITC-labelled streptavidin as the secondary amplification step. The number of positive cells were counted using a fluorescence microscope. Neutralizing activity was defined as present when more than 50% reduction in the number of infected cells relative to control wells was observed, as previously described [20]. Neutralizing activity in plasma diluted ≥1:20 was recorded as a positive result.

### 2.4 Challenge and sampling

Fish in Tank 1 were challenged after 1041 dd, 9 days after transfer to seawater by adding 170 naïve AFC smolts from freshwater in Tank 2, anaesthetized as before and i.p. injected with 0.1 ml of SAV3 inoculum (Isolate 4; Taksdal et al. [10], GenBank LT630447) containing 10^5.1^ TCID_50_/ml. These fish (shedders) represented 20% of the total number of fish in Tank 1 at the start of the challenge period. Dead and terminally weakened moribund fish were removed daily with their PIT-tag identity scanned into the database. A smear from the head kidney of each dead fish was cultured on blood agar with 2% NaCl (BA) and incubated at 22 °C between 48 and 96 h. Evaluation of culture growth was used to detect possible bacterial causes of mortality. Additional samples from dead fish were sent to the Norwegian Veterinary Institute (NVI) for further bacteriological analysis. A small tangential portion of the heart from all dead fish was cut along the sagittal plane and placed into a tube containing RNAlater (Thermo Fischer Scientific, Waltham, MA, USA). Samples in RNAlater were stored overnight at 4 °C and then frozen at −80 °C until use. The RT-qPCR (qPCR) analysis of the heart samples was carried out using a validated and ISO17025 accredited method (Patogen AS, Ålesund, Norway), a probe-based RT-qPCR, as previously described [21]. The cut off Ct value was set to 37. The sampling regime throughout the challenge period is summarized in Table 2.

### 2.5 Viremia

At 19 days post-challenge (dpc), blood was collected, and plasma isolated from fish as detailed in Section 2.3 (also see Table 2) and stored at −80 °C until use. The TCID_50_ end-point titration for the detection and quantification of SAV in plasma samples was performed as previously described [22] with some modifications. In short, the individual plasma samples in ten-fold dilution series were seeded on CHSE-214 cells in 4 replicate wells (96 well plate). After 7 days of incubation at 15 °C, the cell layer was fixed with 80% acetone. SAV-infected cells were visualized using an indirect immunofluorescence test according to the procedure by Falk et al. [18], but with the use of monoclonal antibody 17H23 directed against the E2 glycoprotein of SAV [19] as the primary antibody and with biotin labelled goat anti-mouse Ig and FITC-labelled streptavidin as the secondary amplification step. Standard TCID_50_ end-point titers were determined by microscopic examination and calculations according to Kärber [23].

### 2.6 Histopathology

Fish were sampled for histopathological analysis as outlined in Tables 2 and 3. Formalin-fixed samples of heart, pancreas, red and white skeletal muscle were processed routinely for paraffin embedding. For each fish, a single sagittal section was obtained through the heart, which included ventricle, atrium and bulbus arteriosus. To evaluate the pancreas, a single transverse section was acquired through the pyloric ceca; each of these sections invariably contained multiple islands of exocrine and endocrine pancreatic tissue. Skeletal muscle from the lateral line region was microtomed to provide one transverse section and two longitudinal sections per fish that each contained both red and white skeletal muscle. Histologic sections (4–6 µm thick) were mounted on glass slides and stained with hematoxylin and eosin using standard procedures. All histologic slides were examined via brightfield microscopy at various magnifications (20x – 400x) by an experienced anatomic pathologist, certified by the American College of Veterinary Pathologists. All specimens were examined in a blinded manner, i.e., the pathologist was unaware of the treatment-group status of individual fish. Histopathological changes associated with SAV3 infection post-challenge were recorded for each tissue type separately (i.e., heart, pancreas, red muscle, and white muscle) on a per-fish basis as reported in the parallel study [15] and also detailed in Table 3. Each characteristic was scored for severity using a 0–3 scale as follows: 0 = not remarkable, 1 = mild changes, 2 = moderate changes, 3 = severe changes. Representative pictures and descriptions of the histopathological changes (Grades) for heart, pancreas and muscle applied in this study are available as supplementary Figs. S1, S2 and S3, respectively.

**Table 3:** The semi-quantitative scoring system applied for histopathological evaluation post SAV3 challenge. “n.a.” = not applicable.

**Organ**	**Score**	**Necrosis**	**Inflammation**	**Fibrosis**	**Muscle Regeneration**	**Tissue Loss**
**Heart**	1	1 necrotic myocyte per section to 1 necrotic myocyte per 40x field	1–4 discontinuous layers of epicardial leukocytic infiltrates	Collagen fibers < 10% of muscle tissue	Regeneration < 10% of muscle tissue	n.a.
	2	2 to 4 necrotic myocytes per 40x field	5–10 layers of epicardial leukocytic infiltrates, +/- myocardial infiltrates	Collagen fibers ≥ 10% but ≤ 50% of muscle tissue	Regeneration ≥ 10% but ≤ 50% of muscle tissue	n.a.
	3	> 4 necrotic myocytes per 40x field	> 10 layers of epicardial leukocytic infiltrates, +/- myocardial infiltrates	Collagen fibers > 50% of muscle tissue	Regeneration > 50% of muscle tissue	n.a.
**Skeletal Muscle** **(red and white scored individually)**	1	1 necrotic myocyte per section to 1 necrotic myocyte per 20x field	Leukocytic infiltrates < 10% of muscle tissue	Collagen fibers < 10% of muscle tissue	Regeneration < 10% of muscle tissue	n.a.
	2	2 to 4 necrotic myocytes per 20x field	Leukocytic infiltrates ≥ 10% but ≤ 50% of muscle tissue	Collagen fibers ≥ 10% but ≤ 50% of muscle tissue	Regeneration ≥ 10% but ≤ 50% of muscle tissue	n.a.
	3	> 4 necrotic myocytes per 20x field	Leukocytic infiltrates > 50% of muscle tissue	Collagen fibers > 50% of muscle tissue	Regeneration > 50% of muscle tissue	n.a.
**Exocrine Pancreas**	1	< 10% of acinar tissue necrotic	Leukocytic infiltrates < 10% of pancreatic tissue	Collagen fibers < 10% of acinar tissue	n.a.	< 50% of acinar tissue lost
	2	≥ 10% to ≤ 50% of acinar tissue necrotic	Leukocytic infiltrates ≥ 10% but ≤ 50% of pancreatic tissue	Collagen fibers ≥ 10% but ≤ 50% of acinar tissue	n.a.	≥ 50% of acinar tissue lost, but some acinar tissue remains
	3	> 50% of acinar tissue necrotic	Leukocytic infiltrates > 50% of pancreatic tissue	Collagen fibers > 50% of acinar tissue	n.a.	All acinar tissue lost

### 2.7 Statistical analysis

Initial analyses were undertaken using Pivot tables and graphs in Excel. All further statistical analyses were done using Stata/ MP 15 for Windows (StataCorp, College Station, TX). For the plasma neutralization data, the end titers (<1:20; 1:20; 1:40; 1:160; 1:320) were re-coded into ordinal variables (0; 1; 2; 3; 4), with <1:20 deemed as an absence of neutralizing titer. Differences between groups were statistically verified by tabular analyses (Fisher exact test). An ordinal logistic regression model was used to calculate odds ratios (OR) for the plasma neutralization results under the proportionality assumption using confidence intervals of 95% compared to a reference group. Analysis of the mortality levels between the groups was carried out using Kaplan-Meier failure estimates followed using Cox proportional Hazard regression, where Risk Ratios were estimated as the Hazard Ratio with corresponding 95% Confidence Intervals. The model assumptions were tested using graphical techniques (sthplot). Underlying assumptions of the Cox model were not violated. The qPCR results from the heart tissue of dead fish were analysed using the Kruskal-Wallis and Wilcoxon tests. For statistical analysis of the viremia data, values for TCID_50_ were transformed to log values, and finally into ordinal coding where 0=negative; 1 = 0–10^6^, 2 = 10^6^–10^8^, 3 ≥ 10^8^. Statistical analyses of data were performed using nonparametric methods. Initially a standard rank-based test was used (Kruskal-Wallis test) followed by application of a quantile regression platform using the Saline group as the baseline and then comparing the immunized treatment groups. The results are given as coefficients with 95% confidence intervals and corresponding p-value. Standard ANOVA was used to ascertain whether there were any differences between the groups in the initial weight data. Analyses of weight gain was carried out using a linear regression platform, with the robust standard error estimator. Results are shown as coefficients with 95% confidence intervals and corresponding p-values. The histopathology data were first examined using tabular and graphical techniques followed by ordinal logistic regression analysis. The results are presented as OR with 95% Confidence interval and corresponding p-values. Significance for all tests was established as *p*-value < 0.05 (two sided).

## 3 Results

### 3.1 Growth, side effects and neutralization pre-challenge

**Fig. 1:**
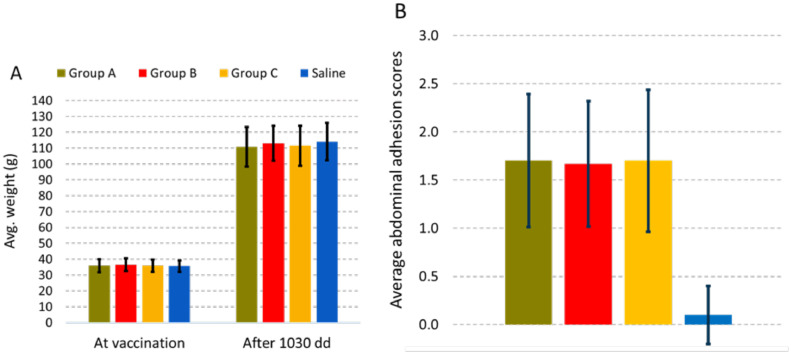
Graph A: Average weights ± one standard deviation of fish sampled at vaccination (*n* = 48–50 per group) and 1030 dd post vaccination with no significant differences in weight gain between the groups based on regression analysis. Graph B: Average abdominal adhesion scores ± one standard deviation of fish sampled 1030 dd post vaccination (*n* = 30 per group) with the Saline group showing significantly lower levels than the vaccinated groups (Fisher exact test *p*<0.001) but no differences between the vaccine groups.

**Fig. 2:**
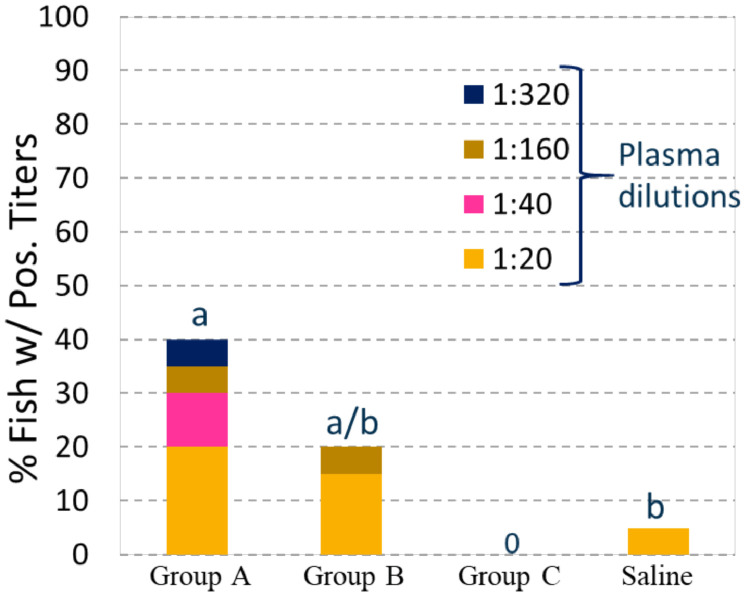
Prevalence and end-titers of SAV3 neutralizing antibodies after immune response period of 1030 dd. The neutralizing activity in plasma from group A was significantly greater than in the Saline group as denoted by different letters (a versus b) (Fisher Exact Test and Ordinal Regression Model *p*<0.05).

The growth of the immunized and saline injected fish in Tank 2 from time of vaccination until smoltification, 1030 dd later, was not significantly different between groups (Fig. 1A). There were no differences in abdominal adhesion scores between the immunized fish groups, but the immunized groups had significantly more adhesions than the Saline group (*p* < 0.001) (Fig. 1B). The levels of abdominal adhesions in the fish immunized with the three OAV's were comparable to those listed their respective SPCs. The prevalence and end-titers of plasma SAV3-neutralizing activity in the non-challenged fish sampled from tank 2 is illustrated in Fig. 2. Plasmas from 40% of fish in Group A (8 of 20) revealed neutralizing capacity with end titers ranging from 1:20 to 1:320. This was significantly higher than the Saline group, where only one fish (5%) had an end-titer of 1:20. In comparison, 20% of fish in group B (4 of 20 fish) had neutralizing end-titers ranging from 1:20 to 1:40, which were not significantly different from those in group A or the Saline group. No neutralizing activity was measured in the plasmas of group C.

### 3.2 Mortality

**Fig. 3:**
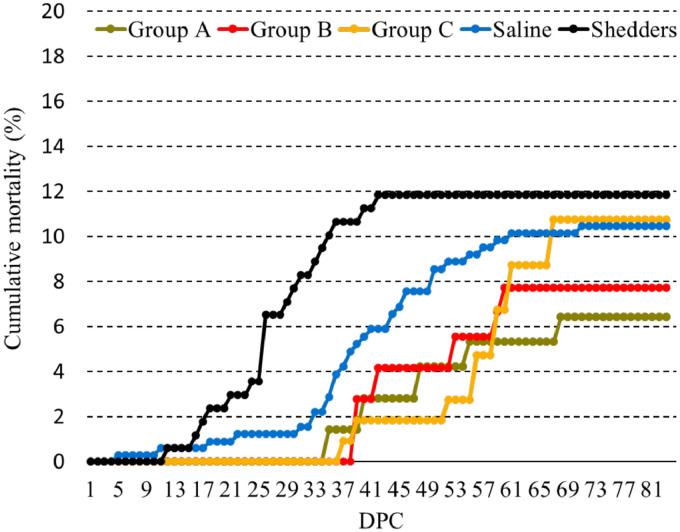
Cumulative% mortality post challenge. No significant differences were found between any of the vaccine groups compared to the Saline group although group A came closest (Cox regression *p* = 0.11).

Mortality during the immune response period prior to challenge was minimal (<0.9%) and not associated with infectious disease. Mortality levels post challenge were low, reaching 10.4% in the Saline group (Fig. 3), which was comparable to mortality in the SAV3 injected shedder fish (11.8%). Fish in group A experienced the lowest mortality, 6.4%, but this was not statistically significantly different (*p* = 0.11) from the Saline group. During the challenge period, mortality in the Saline group occurred between 5 and 71 dpc while fish in the immunized groups died between days 35 and 68 dpc.

**Fig. 4:**
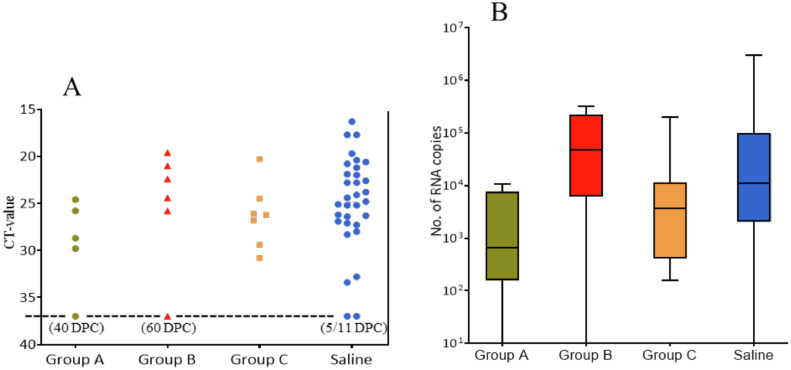
Graph A: qPCR SAV3 results showing individual Ct-values from hearts of all dead fish. The dotted line denotes the negative cut-off value of 37 and days post challenge (DPC) for each of the Ct-negative hearts. Graph B: Box plot showing number of SAV3 RNA copies calculated using the Ct-values as previously described [21]. The lines across each box depict the median for each group. No statistical differences in number of SAV3 RNA copies were found between the groups.

The SAV3 qPCR results of heart samples from the dead fish revealed highly variable Ct-values within and between groups. Of the 32 fish that died in the Saline group, 30 fish (94%) were positive by qPCR (Ct-values ranging from 16.3 to 24.1), which indicated that horizontal SAV3 transmission was successful. Of the fish in groups A, B and C that died, 80% (4 of 5 fish), 83% (5 of 6 fish) and 100% (7 of 7 fish) were similarly positive by qPCR, respectively (Fig. 4A). By transcribing these Ct-values into estimated number of viral RNA copies, as previously described for this qPCR procedure and primer set [21], no significant differences were found in amount of SAV3 RNA between any of the groups (Fig. 4B).

The majority of dead and moribund fish in the challenge tank, irrespective of the treatment group, had skin ulcers. Proportions of dead fish with skin ulcers was 5 of 5 (100%), 6 of 6 (100%), 7 of 8 (88%) and 23 of 34 (68%) in groups A, B, C the Saline, respectively. The prevalence of skin ulcers in surviving fish was lower than in dead fish, and similar across treatment groups, but increased with time ranging from 0 to 5%, 20–30%, and 29–41% at 19, 54 and 83 dpc, respectively. Cultures from the head kidney of dead fish on blood agar plates revealed sparse to moderate growth of mixed bacterial colonies. Pathogenic bacteria known to be associated with skin ulcers in seawater-reared Atlantic salmon, *Moritella viscosa* and *Tenacibaculum* spp., were not identified. Moribund fish were commonly observed rubbing against the bottom of the tank, which may have given access for opportunistic bacteria to cause the increased prevalence of skin ulcers in dead and moribund fish compared to surviving, sampled fish.

### 3.3 Viremia

**Fig. 5:**
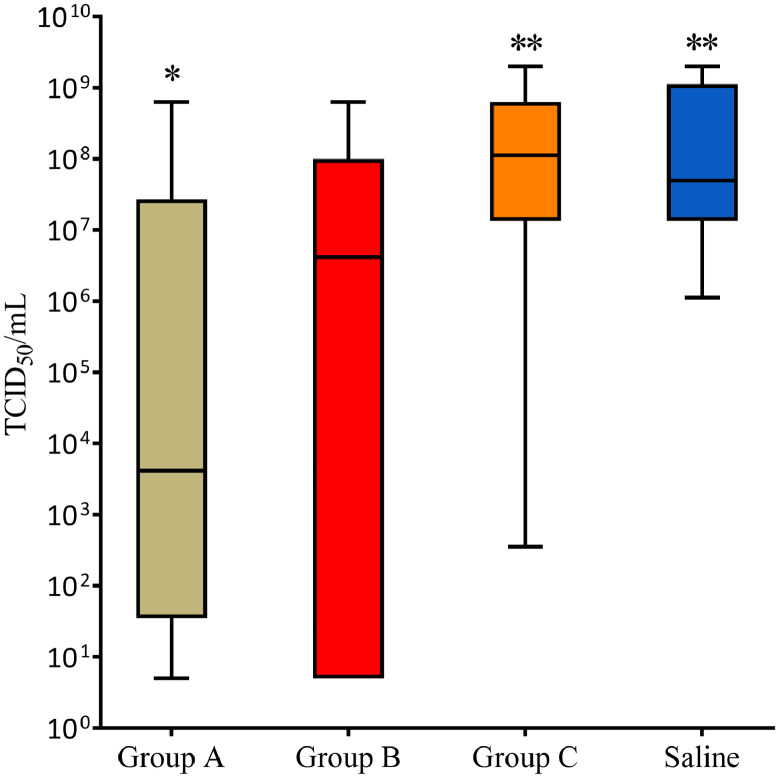
Box plot showing SAV3 viremia (TCID50/mL) of groups at 19 dpc. Group A had significantly less virus in plasma than the groups C and Saline denoted with asterisks * versus ** (*p*<0.01).

The SAV3 virus loads measured in plasma at 19 dpc are shown in Fig. 5. There was larger variation in plasma virus titers of individual fish within the immunized groups A and B compared to group C and Saline. Fish in group A had significantly lower SAV3 titers than those in group C and the Saline group (*p* < 0.01). Titers in group B were not significant different compared to any of the other groups.

### 3.4 Weight gain

**Fig. 6:**
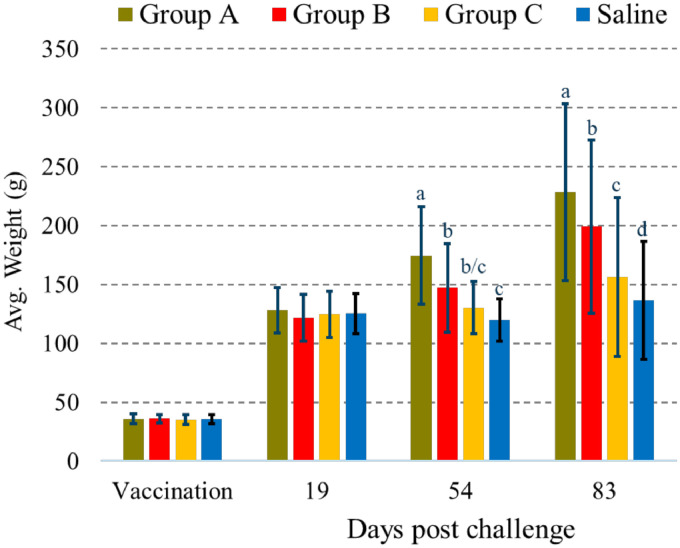
Average weights ± one standard deviation of fish sampled at vaccination (*n* = 112–115 for groups A-C; *n* = 341 for Saline), 19 (*n* = 20), 54 (*n* = 20) and 83 dpc. At 83 dpc, the remaining 65, 65, 62 and 262 fish were weighed from groups A, B, C and Saline, respectively. Different letters (a, b, c or d) denote statistically significant differences in weight gains between the groups within each sampling point (Linear regression analysis *p*<0.05).

Based on the standard ANOVA analysis, there were no differences in the initial lengths (*p* = 0.18) and weights (*p* = 0.27) of fish among the groups at the time of vaccination. Correlation between recorded length and weight increases were high (0.87), which indicated that the two parameters increased correspondingly, and thus provided similar information. Therefore, only weights are presented (Fig. 1). At both 54 and 83 dpc, fish in group A had gained statistically significantly greater weight than the other groups (Fig. 6) while fish in group B had gained significantly more weight than Saline at 54 dpc, and significantly more weight than group C and Saline at 83 dpc. Fish in group C gained significantly greater weight than Saline, but only at the 83 dpc timepoint. In contrast to fish in groups A and B, no weight gain was registered in group C and Saline between 19 and 54 dpc, while all fish groups gained weight between 54 and 83 dpc.

### 3.5 Histopathology

**Fig. 7:**
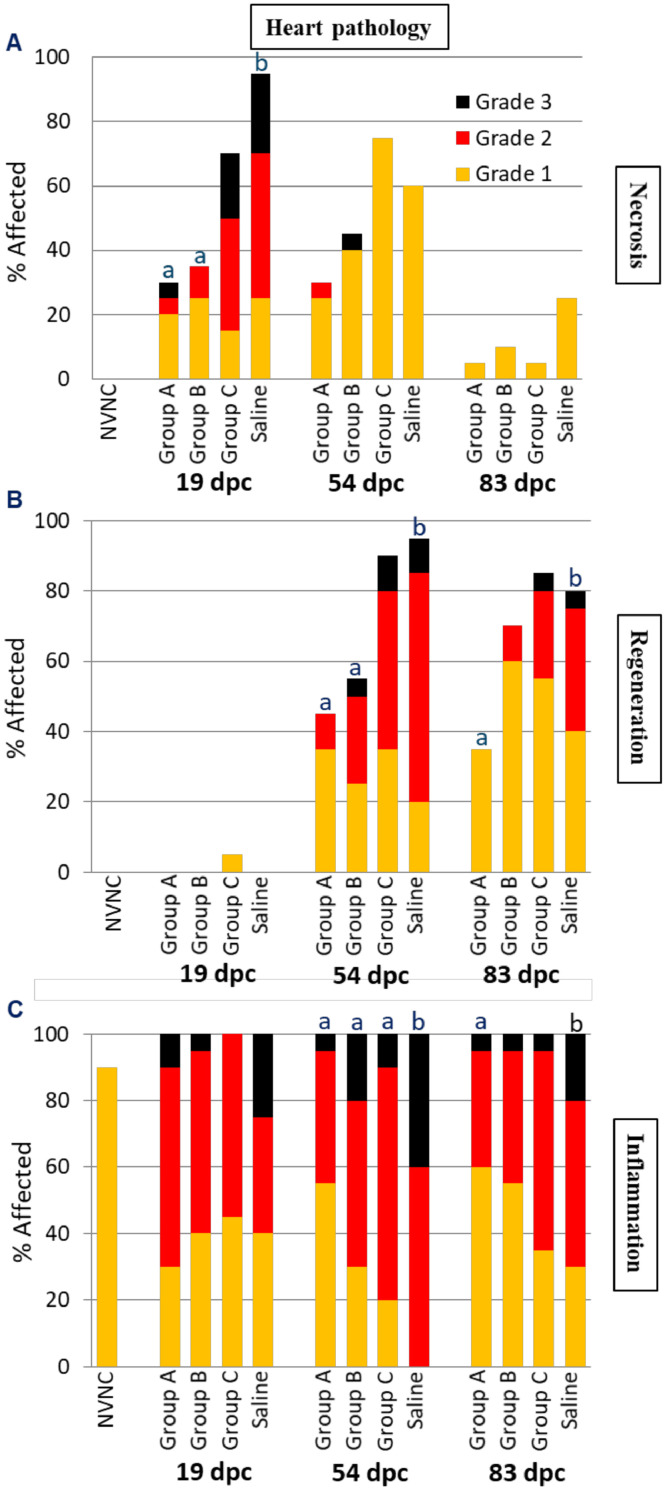
The prevalence and severity of necrosis (A), cardiac myocyte regeneration (B), and inflammation (C) in hearts sampled at 19, 54 and 83 dpc (*n* = 20 per group). NVNC indicates non-vaccinated and non-challenged controls. Different letters (a and b) denote statistically significant differences between the groups within each pathology criterium each sampling point (Ordinal logistic regression *p*<0.05) with NVNC excluded.

The results from the histopathological analysis of the hearts are illustrated in Fig. 7. The highest prevalence and severity of cardiac necrosis occurred at 19 dpc, which in all groups was followed by a general decline in this finding at 54 and 83 dpc, respectively (Fig. 7A). At 19 dpc, only fish in groups A and B had significantly less cardiac necrosis compared to the Saline group, whereas no such differences were registered at 54 and 83 dpc, where the overall degree of necrosis was very low. No heart necrosis was registered in the NVNC fish. In affected fish, necrotic myocytes were shrunken with pale eosinophilic cytoplasm, irregular cytoplasmic margins and nuclei that were pyknotic, karyorrhectic, or absent (ghost nuclei); this appearance was consistent with coagulative necrosis. Occasional necrotic myocytes were small and rounded with pyknotic nuclei, and were surrounded by a clear halo, consistent with apoptotic necrosis.

Cardiac myocyte regeneration was completely absent at 19 dpc. The prevalence and severity of this finding appeared to peak at 54 dpc and fell off slightly by 83 dpc (Fig. 7B). While fish in groups A and B had significantly less cardiac myocyte regeneration compared to the Saline group at 54 dpc, only group A retained this difference at 83 dpc. Fish in group C were not significantly different from the Saline group at either time point. Regeneration was characterized by patchy areas of myocytes that had hypertrophic (enlarged) “open-faced” nuclei, clumped and peripheralized heterochromatin, prominent nucleoli, and decreased amounts of sarcoplasm which was slightly basophilic. Regeneration was most often observed at the interface between the cardiac stratum compactum and stratum spongiosum. Although the process of regeneration is considered beneficial to the healing process, a higher regeneration score suggests a greater degree of initial heart damage and was therefore considered a negative health indicator in this study. As expected, NVNC fish did not exhibit any cardiac myocyte regeneration.

A very high prevalence (≥ 90%) of cardiac inflammation Grade ≥1was observed in all the OAV groups and Saline (NVNC fish included) at all sampling points (Fig. 7C). Unlike the treated groups, no fish in the NVNC group displayed Grade 2 cardiac inflammation. The severity of cardiac inflammation was lowest at 19 dpc, at which point there were no significant differences between the groups. At 54 dpc and with exception of fish in group A, the severity of cardiac inflammation had increased in all groups and mostly in the Saline group, rendering it significantly greater than the three immunized groups. At 83 dpc, the severity of cardiac inflammation had decreased in all groups compared to 54 dpc, with only fish in group A showing significantly reduced severity compared to the Saline group. The inflammatory cell infiltrates consisted predominantly of lymphocytes, fewer non-lymphocytic mononuclear cells, and occasional eosinophilic granular cells. Inflammation was primarily epicardial and generally limited to the ventricle, although lesser degrees of ventricular myocardial inflammation were occasionally evident, especially in hearts that received scores of Grades 2 or 3.

**Fig. 8:**
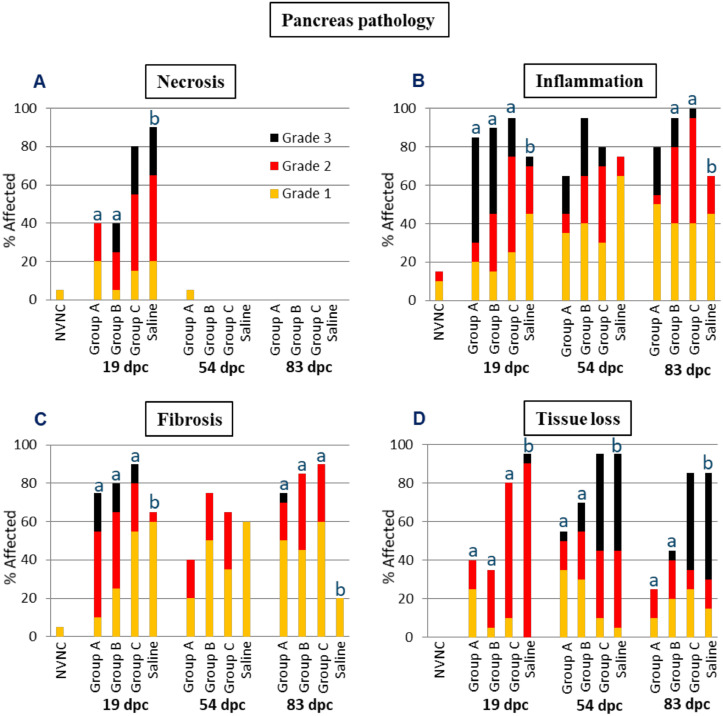
The prevalence and severity of necrosis (A), inflammation (B), fibrosis (C) and tissue loss (D) of the pancreas sampled at 19, 54 and 83 dpc (*n* = 20 per group). NVNC indicates non-vaccinated and non-challenged controls. Different letters (a and b) denote statistically significant differences between the groups within each pathology criterium and sampling point (Ordinal logistic regression *p*<0.05) with NVNC excluded.

The results from the pancreas pathology analysis are illustrated in Fig. 8. Most of the pancreatic necrosis was observed only at 19 dpc (Fig. 8A), and the prevalence and severity of necrosis was significantly lower levels in groups A and B compared to the Saline group. In contrast, the degree of pancreatic necrosis in group C was not significantly different from the Saline group. Necrosis was characterized by apoptotic fragmentation of acinar cells and the presence of cellular debris, all of which spared the endocrine pancreas. The limitation of pancreatic necrosis to 19 dpc is consistent with results from previous studies [13,15,24,25].

The prevalence if pancreatic inflammation remained relatively high at all sampling points. The severity of pancreatic inflammation was highest at 19 dpc and was followed by a gradual decrease at the latter two time points, particularly in the Saline group, while severity in the immunized groups remained relatively higher (Fig. 7B). At 19 dpc, all the immunized groups had significantly greater pancreatic inflammation compared to the Saline group. At 54 dpc, none of the immunized groups had significantly different degrees of pancreatic inflammation as compared to the Saline group. At 83 dpc, groups B and C showed significantly greater pancreatic inflammation compared to the Saline group while group A did not. A low degree of background pancreatic inflammation was found in two NVNC fish. Pancreatic inflammation was dominated by lymphocytes and non-lymphocytic mononuclear cells, with fewer neutrophils and only occasional eosinophilic granulocytes.

Except for the fish in group A at 54 dpc, prevalence and severity levels of pancreatic fibrosis were higher in the immunized groups than the Saline group at all time points (Fig. 8C). At 19 dpc, the fish in the immunized groups all had significantly greater levels of pancreatic fibrosis than the Saline group. At 54 dpc, the fish in all the groups showed reduced  pancreatic fibrosis compared to 19 dpc and with no significant difference among the groups. At 83 dpc, the pancreatic fibrosis levels were again significantly higher in all the immunized groups compared to the Saline group. A single NVNC fish exhibited Grade 1 pancreatic fibrosis. Fibrosis was characterized by variably sized, moderately cellular sheets of immature collagenous connective tissue that were spatially associated with low levels of inflammation in some samples.

While the prevalence of pancreatic tissue loss remained relatively constant at all sampling points, the severity of this condition progressed with time, particularly in groups C and Saline (Fig. 8D). With all the immunized groups demonstrating significantly reduced pancreatic tissue loss compared to the Saline group at 19 dpc, such difference where limited to groups A and B at the latter two sampling points. Most fish in groups C and Saline had complete (Grade 3) tissue loss at both 54 and 83 dpc. No NVNC fish exhibited pancreatic tissue loss. Tissue loss was characterized by the partial or complete absence of exocrine acinar cells.

**Fig. 9:**
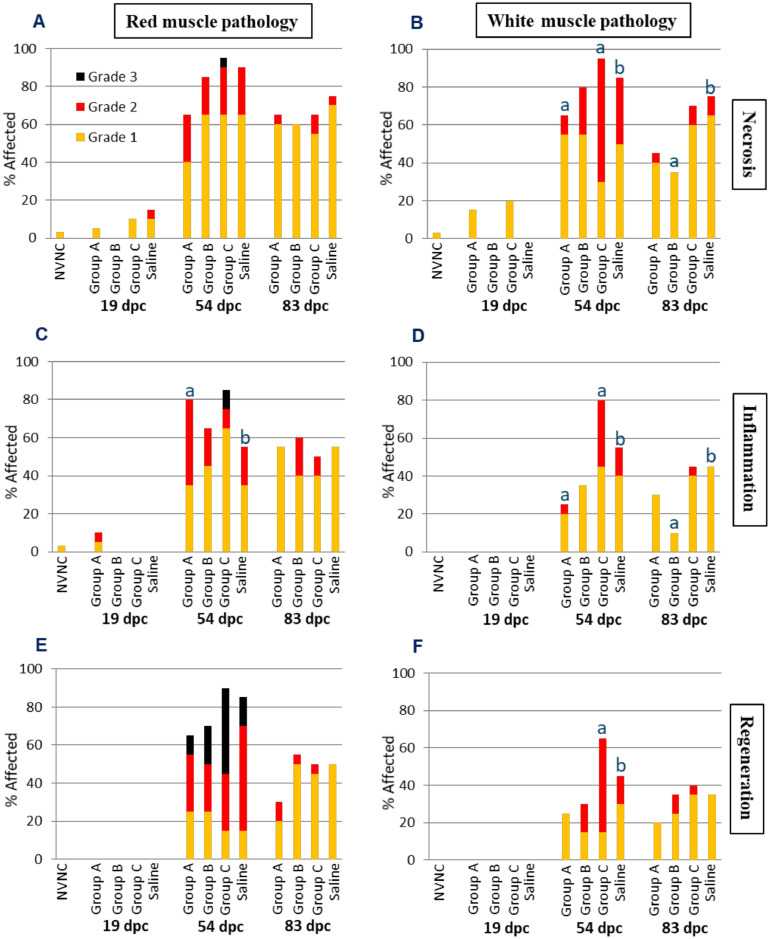
The graphs illustrate the prevalence and severity of red and white muscle necrosis (A and B), inflammation (C and D) and regeneration (E and F) sampled at 19, 54 and 83 dpc (*n* = 20 per group). NVNC indicates non-vaccinated and non-challenged controls. Different letters (a and b) denote statistically significant differences between the groups within each pathology criterium and sampling point (Ordinal logistic regression *p*<0.05) with NVNC excluded.

The results from the red and white muscle pathology analysis are illustrated in Fig. 9. At 19 dpc, there was negligible red or white muscle necrosis in all groups. The prevalence of both red and white muscle necrosis peaked in all the groups at 54 dpc. No significant differences in red muscle necrosis levels were found between the groups at 54 or 83 dpc. There was significantly less and significantly greater white muscle necrosis, respectively, in groups A and C at 54 dpc as compared to the Saline group, while group B was not significantly different. At 83 dpc, only group B exhibited significantly different (lower) white muscle necrosis compared to Saline (Fig. 9A and B). Only one NVNC fish exhibited Grade 1 red and white muscle necrosis. Skeletal muscle necrosis was characterized by individual myofibers that were fragmented and hypereosinophilic with loss of striations, and necrotic myofibers were frequently accompanied by proliferating peripheral and internalized uninuclear satellite cells.

The severity of red and white muscle inflammation was generally low, i.e., predominantly Grade 1 (Fig. 9C and D).  The occurrence of inflammation generally corresponded to the degree of necrosis, and thus little inflammation was evident until the peak 54 dpc, after which it largely subsided. At 54 dpc, only group A had significantly greater red muscle inflammation than the Saline group, while no significant differences were found between the groups at 83 dpc (Fig. 9C). At 54 dpc, groups A and C, respectively, exhibited significantly lower and greater white muscle inflammation than the Saline group. At 83 dpc, only group B showed significantly different (lower) inflammation compared to the Saline group (Fig. 9D). Inflammatory cell infiltrates in skeletal muscle were comprised almost exclusively of lymphocytic and non-lymphocytic mononuclear cells.

The magnitude of red and white muscle regeneration was moderate and low, respectively (Fig. 9E and F). The prevalence of muscle regeneration closely mirrored the prevalence of inflammation and fibrosis (data not shown). There was essentially no muscle regeneration at 19 dpc. The prevalence and severity of regeneration peaked at 54 dpc in both muscle types prior to considerable reduction by 83 dpc. No significant differences in muscle regeneration were found between any of the groups at any time point except for group C showing significantly greater white muscle regeneration than the Saline group at 54 dpc. No muscle regeneration was observed in the NVNC control group. Regeneration was characterized by the presence of narrow serpentine myofibers with hypertrophic nuclei and basophilic cytoplasm. The severity of red and white muscle fibrosis was generally low. The only difference in muscle fibrosis occurred at 54 dpc, at which point white muscle fibrosis in group A was significantly less than in the Saline group (data not shown).

## 4 Discussion

In this study, only the DNAV immunized fish (group A) demonstrated a significant increase in virus neutralization titer towards SAV3 compared to the Saline control group. Although neutralizing antibody activity, as measured *in vitro*, may contribute to protect against PD [15,26,27], the relative contribution is unknown, and cellular adaptive immune responses are believed to play an integral and complementary role [28]. The neutralizing titer in plasma warrants further investigation as a potential biomarker, especially since this parameter correlated to the other measured efficacy criteria, except mortality. The role of neutralizing titers is also of interest as high plasma titers have been observed in Atlantic salmon that have recovered from natural PD outbreaks [3,17,29].

At 19 dpc, viremia levels were significantly lower in group A compared to groups C and Saline but not compared to group B. These results corresponded with the scoring of the cardiac necrosis at 19 dpc, when only groups A and B exhibited significantly less necrosis compared to Saline. In the parallel study where the fish was immunized with the DNAV only, significantly lower viremia levels were also measured [15], and similarly found in fish that were immunized with an experimental SAV3 DNA vaccine [27]. In earlier SAV3 challenge studies performed at similar temperatures as the present experiment, viremia was estimated to peak between 4 and 13 dpc [10,25], i.e.*,* considerably earlier than 3 weeks post-challenge as found in this study. This difference is likely due to the i.p. challenge that were used in the previous studies, while cohabitation using shedder fish was used in the present study. Cohabitation mimics more closely the natural route of infection where the virus first needs to pass mucosal barriers. Based on this information, sampling at 19 dpc was chosen to represent the expected peak of viremia, although the precise timing of the viremia peak was not determined. Viremia peaking at different timepoints between groups based on varying susceptibility to the virus cannot be ruled out. Nevertheless, the high viremia levels in groups C and Saline at 19 dpc suggest that the timing of sampling was appropriate.

Important clinical manifestations of PD include appetite loss and reduced growth rate [10,15,30,31]. Growth reduction caused by SAV3 has earlier been identified as the most important factor for the economic impact of PD in Norway [14]. The post-infection growth data in this study showed that fish in group A were significantly better protected against PD-induced weight loss than the other groups at 54 and 83 dpc. At 83 dpc, all the immunized groups had experienced significantly greater weight gain than the Saline group in the order group *A* > group *B* > group C. No differences were found in weight gain between the groups at 19 dpc which at this time-point corresponded with the weight gain of the non-challenged group in tank 2, and thus supported the notion that different weight gains observed at later time points post challenge were due to variations in the level of protection against PD between the groups. The difference in post-challenge weight gain between groups A and B in this study corresponds with results from a comparative field efficacy study comparing the same vaccines within the same rearing units under normal farming conditions [32].

The cumulative mortality of 10.4% in the Saline group was similar to that observed in previous cohabitation challenges using the same SAV3 isolate [10,15]. The cumulative mortality in groups A and B was lower, but not significantly lower, than the mortalities in the groups Saline and C. The high prevalence of skin ulcers in the dead and moribund fish irrespective of treatment group, was also observed in the previous SAV3 challenge study where the fish had been immunized with DNAV only [15], and similarly, the etiology of the ulcers was not revealed by bacteriological examination. The very high prevalence of skin ulcers cannot, however, be excluded as a contributing factor to the mortality. Based on lower prevalence of skin ulcers in the surviving sampled fish compared to dead and moribund fish and lack of detectable pathogenic bacteria in the skin ulcers, together indicate that the skin ulcers appear as secondary complication to the primary PD infection. The increase in prevalence of skin ulcer with time after virus exposure indicated that the susceptibility of the fish to develop skin ulcers corresponded with prevalence and severity of pancreatic tissue loss.

The reason for the high prevalence of cardiac inflammation Grade ≥1 observed in ≥ 90% all the fish groups at all sampling points (NVNC fish included) can be attributed to the low threshold used for this diagnosis, e.g. a single focus of 3–5 mononuclear cells along the epicardial surface would trigger a diagnosis of Grade 1 inflammation. Low numbers of epicardial mononuclear leukocytes likely represent normal hematopoietic tissue [33], but such cells are difficult to distinguish from inflammatory leukocytes in histologic sections. The time-course and characteristics of histopathologic findings in the heart, pancreas, and skeletal muscle were generally consistent with those of previous studies of SAV3-induced changes [10,15,24,34]. Based on the relative severity of these changes, the fish in groups A and B were significantly better protected against SAV3-induced disease in the heart, pancreas, and skeletal muscle when compared with group C or Saline. However, the severity and prevalence of visceral pancreatic inflammation and fibrosis remained higher in vaccinated fish than in Saline control throughout the challenge period. These results suggest that the increased inflammation and fibrosis are attributable to an enhanced localized immunologic response associated with the intraperitoneal injection of the multivalent OAVs, as opposed to direct pathologic effects of SAV3 infection. This hypothesis is also consistent with the apoptotic appearance of pancreatic necrosis in SAV3-infected fish, as the process of apoptosis itself is not thought to elicit a substantial inflammatory response [35].

As previously suggested [36], there are a few ways by which tissue damage attributable to SAV3 infection could impact weight gain and growth. First, damage to cardiac and skeletal muscles may hinder the swimming performance of affected fish and thus decrease their ability to compete for feed. Second, the multi-organ inflammatory response associated with the viral infection and tissue necrosis may contribute to general malaise and appetite loss. Third, and perhaps most importantly, the loss of pancreatic acinar tissue and corresponding digestive enzyme secretion may impact nutrient absorption and thus inhibit the conversion of feed to energy required for growth. It is noteworthy that while heart and skeletal muscle lesions were in the process of resolving in all groups by 83 dpc, a substantial proportion of fish in group C and Saline control still had Grade 2 or 3 (i.e., 60–70%) pancreatic tissue loss at that time point.

## 5 Conclusions

In the present study, a SAV3 cohabitation challenge run in seawater was employed to evaluate and compare the effect of three vaccination strategies against PD available to the Norwegian salmon farming industry. Only the DNAV immunized fish demonstrated significantly higher virus neutralization titer against SAV3 and significantly lower viremia levels compared to the Saline group. Furthermore, the protection against growth impairment caused by PD was significantly greater in the DNAV immunized fish compared to the other groups at 54 and 83 dpc. This correlated with the lower pancreas tissue loss levels at 54 and 83 dpc in the DNAV immunized fish although this was not significantly lower than the second ranked fish in group B. The only efficacy criterium that was not significantly different between the groups was the mortality, however it was quite low, as reported both in earlier SAV cohabitation challenge experiments [10,15] and commercial farming [9,31,32,37]. In commercial production, the PD vaccines used in this study are normally administered together with multivalent OAVs. The application of these PD vaccines will in due course provide insights into how the results obtained in this study correspond with those obtained in Atlantic salmon farmed in the enzootic PD areas.

## Declaration of Competing Interest

Ragnar Thorarinsson is employed by Elanco Animal Health which funded this study and is the marketing authorization holder of the DNAV. The remaining authors declare that they have no known competing financial interests or personal relationships that could have appeared to influence the work reported in this paper.

## Appendix Supplementary materials

Image, application 1
