# Supplementary material for: Effects of a DNA and multivalent oil-adjuvanted vaccines against pancreas disease in Atlantic salmon (Salmo salar) challenged with salmonid alphavirus subtype 3
Source: Fish Shellfish Immunol Rep. 2022 Aug 10;3:100063. doi: 10.1016/j.fsirep.2022.100063 (PMC9680106; doi:10.1016/j.fsirep.2022.100063)
Supplement: Supplementary file 1 [file mmc1.docx]

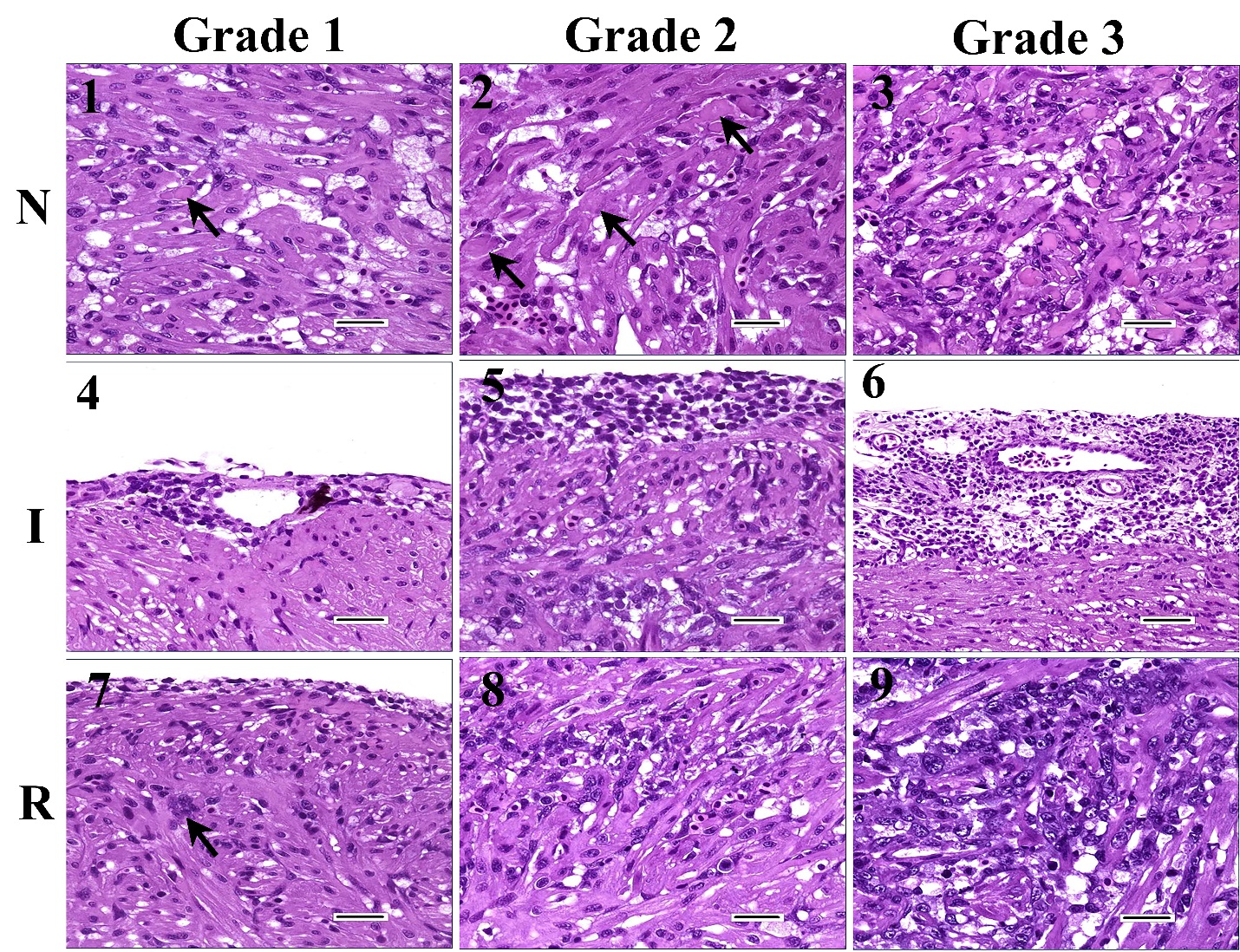


**Fig. S1** Severity grading examples of heart findings. 1) Arrow indicates a single small pale irregularly-shaped necrotic myocyte in the cardiac ventricle. 2) Arrows indicate several necrotic myocytes within a high magnification field. 3) The vast majority of myocytes throughout the ventricle are necrotic. 4) There is a small focus of ventricular epicardial mononuclear cell infiltrates. 5) Epicardial infiltrates form a discontinuous layer approximately 3-9 cells deep. 6) Epicardial infiltrates form a continuous layer, frequently 10-20 cells deep, and myocardial infiltrates are also common. 7) Arrow indicates a single focus of regenerating cardiac myocytes, observed as a small cluster of large basophilic nuclei, within the ventricle at the interface between the stratum compactum and stratum spongiosum. 8) Grade 2 is characterized by multiple focally extensive areas of myocyte regeneration. 9) Myocyte regeneration is widespread throughout the ventricle. N = necrosis, I = inflammation, R = regeneration. Bar sizes: images 1-2, 4-5, and 7-9, bar = 25 mm; images 3 and 6, bar = 50 mm.


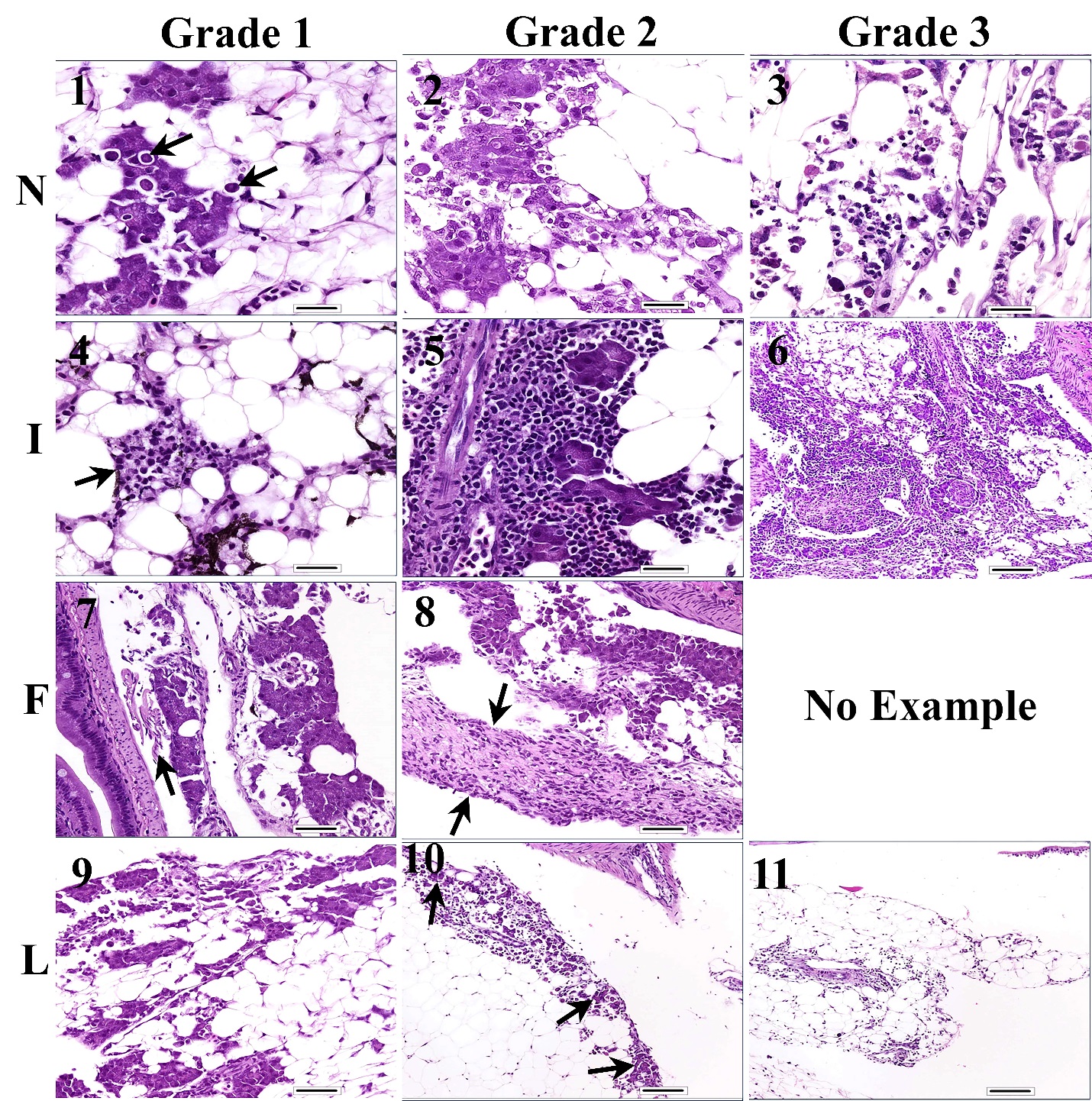


**Fig. S2.** Severity grading examples of exocrine pancreas findings. 1) Arrows indicate single cell necrosis (apoptotic-type) of acinar cells. 2) Necrosis is more widespread, but some of the acinar tissue is spared. 3) Essentially all the acinar tissue is necrotic. 4) A small focus of mononuclear cell infiltrates resides within the peri-pancreatic adipose tissue. 5) Focally extensive area of mononuclear cell infiltration. 6) Widespread inflammation involving the pancreas and adjacent mesenteric adipose tissue. 7) A small focus of several immature collagenous fibers. 8) Larger patchy area of immature fibrous connective tissue (Grade 3 fibrosis was not observed in this study). 9) More than half the exocrine pancreas remains, the rest is necrotic. 10) Less than half the exocrine pancreas remains, but some intact acini (arrows) are still evident. 11) No intact acinar tissue remains. N = necrosis, I = inflammation, F = fibrosis, L = loss of acinar tissue. Bar sizes: images 1-5, bar = 25 mm; images 7-9, bar = 50 mm; images 6 and 10-11, bar = 100 mm.

**
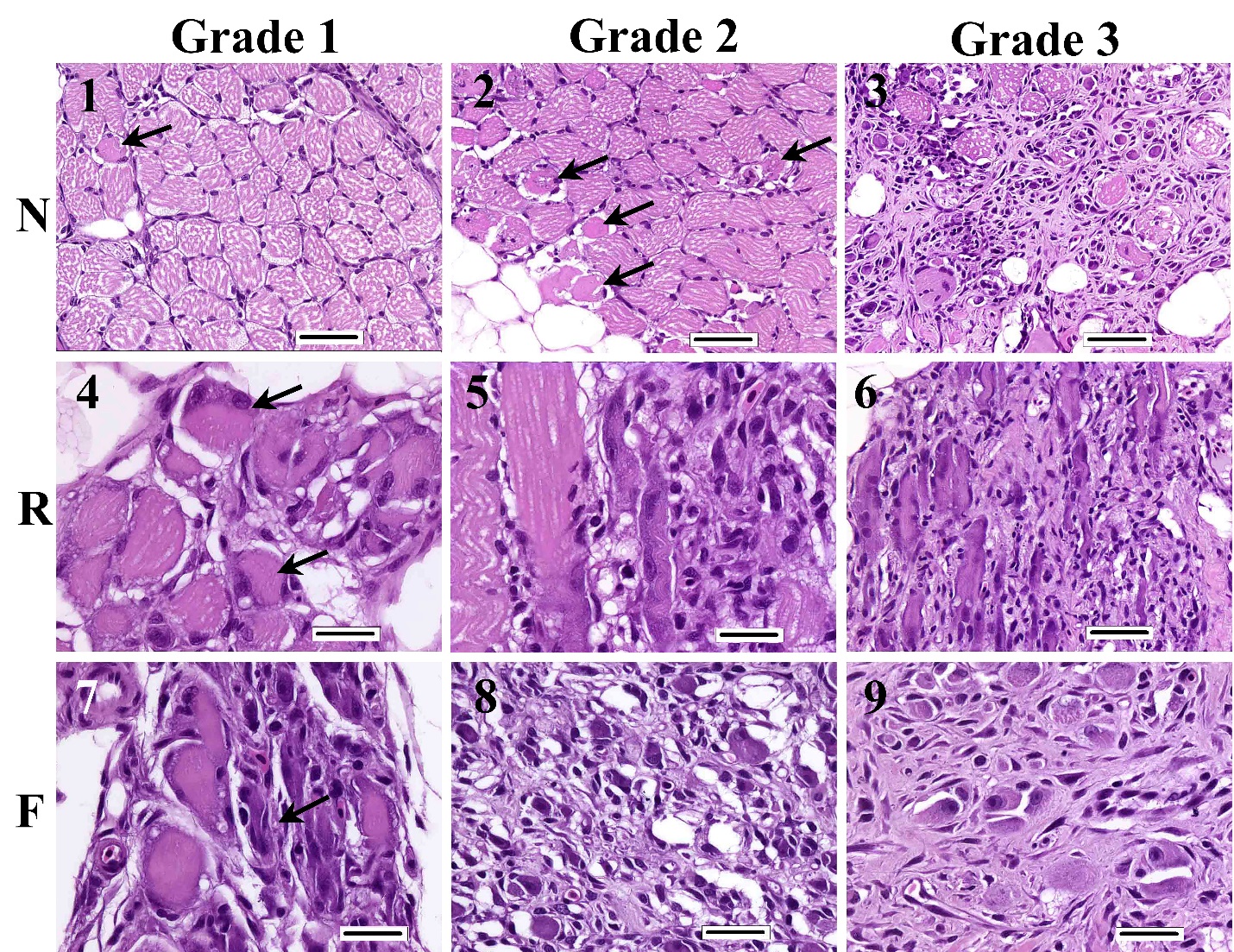
**

**Fig. S3.** Severity grading examples of red muscle findings. 1) Arrow indicates a single necrotic myocyte. 2) Arrows indicate several necrotic myocytes in a high magnification field. 3) Essentially all remaining myocytes in this image are necrotic. 4) A couple of myofibers (arrows) have plump basophilic satellite cell nuclei that are beginning to internalize. 5) Myocyte regeneration is widespread and characterized by elongated multinucleated myofibers, but a couple of unaffected fibers (left) in this image are still evident. 6) Essentially all myofibers in this image are undergoing regeneration. 7) A few small strands of collagenous fibers (arrow) and proliferating fibroblasts can be seen between regenerating myofibers. 8) In patchy areas, regenerating myofibers are diffusely separated by loose fibrous connective tissue. 9) Much of the skeletal muscle is replaced by dense fibrous connective tissue. Severity grading criteria for white muscle were similar to those for red muscle, and are therefore not included. N = necrosis, R = regeneration, F = fibrosis. Bar sizes: images 4-5 and 7-9, bar = 25 mm; images 1-3 and 6, bar = 50 mm.
